# Misregulation of Wnt Signaling Pathways at the Plasma Membrane in Brain and Metabolic Diseases

**DOI:** 10.3390/membranes11110844

**Published:** 2021-10-29

**Authors:** Mustafa Karabicici, Yagmur Azbazdar, Evin Iscan, Gunes Ozhan

**Affiliations:** 1 Izmir Biomedicine and Genome Center (IBG), Dokuz Eylul University Health Campus, Inciralti-Balcova, Izmir 35340, Turkey; mustafa.karabicici@msfr.ibg.edu.tr (M.K.); yagmur.azbazdar@msfr.ibg.edu.tr (Y.A.); evin.iscan@ibg.edu.tr (E.I.); 2 Izmir International Biomedicine and Genome Institute (IBG-Izmir), Dokuz Eylul University, Inciralti-Balcova, Izmir 35340, Turkey

**Keywords:** Wnt signaling pathway, plasma membrane, ordered domain, lipid raft, Alzheimer’s disease, Parkinson’s disease, Schizophrenia, diabetes, obesity, nonalcoholic fatty liver disease, nonalcoholic steatohepatitis

## Abstract

Wnt signaling pathways constitute a group of signal transduction pathways that direct many physiological processes, such as development, growth, and differentiation. Dysregulation of these pathways is thus associated with many pathological processes, including neurodegenerative diseases, metabolic disorders, and cancer. At the same time, alterations are observed in plasma membrane compositions, lipid organizations, and ordered membrane domains in brain and metabolic diseases that are associated with Wnt signaling pathway activation. Here, we discuss the relationships between plasma membrane components—specifically ligands, (co) receptors, and extracellular or membrane-associated modulators—to activate Wnt pathways in several brain and metabolic diseases. Thus, the Wnt–receptor complex can be targeted based on the composition and organization of the plasma membrane, in order to develop effective targeted therapy drugs.

## 1. Introduction

Wnt signaling pathways are highly conserved in the animal kingdom, based on their components and functional roles in the regulation of development, tissue homeostasis, and regeneration [1,2,3,4,5,6,7,8]. Thus, it is not surprising that changes in Wnt pathway components and modulators—including loss or gain of function—play a role in many pathologies associated with growth, development, and cancer. Although major pathway components have been characterized in detail, misregulation of Wnt signaling within the context of human diseases is extremely complex, and remains only partially understood. Understanding of this underlying complexity will enable the identification of novel therapeutic targets for many diseases associated with the Wnt pathway [9,10,11]. 

The plasma membrane plays a fundamental role in the regulation of cell signaling. Regulation occurs through the surface receptors, modulators, and associated lipids that actively control the transmission of molecular signals from the outside to the inside and activate downstream signaling events. The plasma membrane consists of nanodomains—the so-called ordered membrane domains or lipid rafts that are defined as dynamic assemblies of various saturated lipids, sterols, glycosphingolipids, and glycosyl-phosphatidylinositol (GPI)-anchored proteins [12,13,14]. These domains influence membrane fluidity and receptor trafficking, thereby playing a key role in the functioning of receptors, protein sorting, and regulation of receptor-mediated signaling [15,16,17,18]. These nanodomains have been revealed to be altered in various diseases, including cancer, neurological and neurodegenerative diseases, and metabolic diseases [19,20,21]. Changes in the composition and organization of membrane proteins and lipids also play an important role in Wnt pathway activation and, thus, in the pathology of pathway-associated diseases [22,23]. Considering that the membrane proteins account for over 60% of the targets of all FDA-approved small-molecule drugs, it is critical to characterize Wnt pathway components that act across the plasma membrane as potential therapeutic targets [9,22,24,25]. Here, we review the abnormal regulation of the Wnt signaling pathway in brain and metabolic disorders. In particular, we address how plasma membrane components of Wnt pathways and membrane domain organization are affected in Alzheimer’s disease (AD), Parkinson’s disease (PD), Schizophrenia (SZ), diabetes, obesity, nonalcoholic fatty liver disease (NAFLD), and nonalcoholic steatohepatitis (NASH).

## 2. Wnt Signaling Pathways

**Figure 1:**
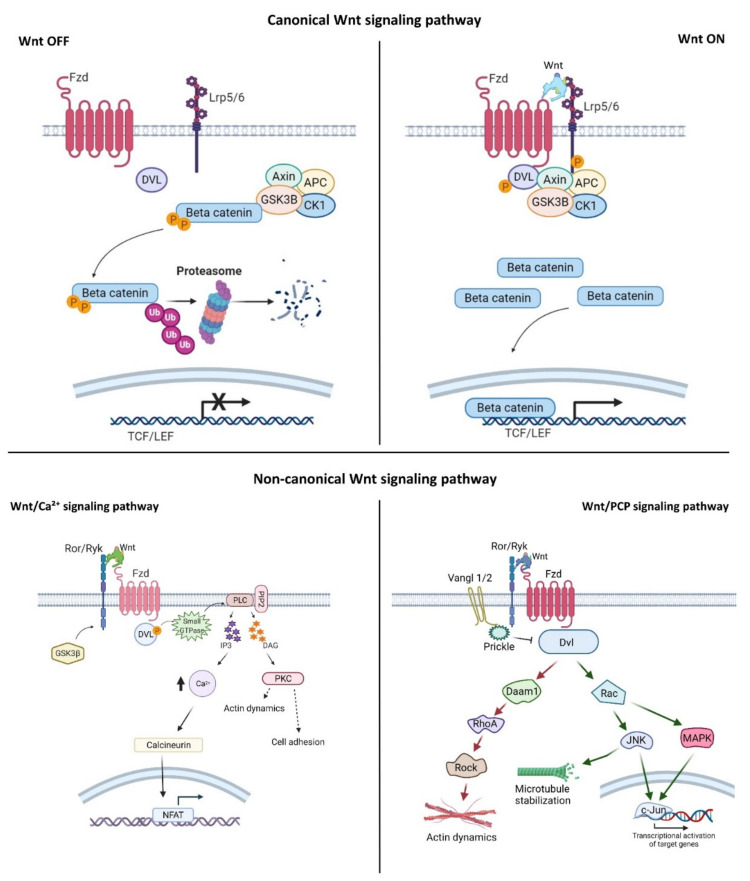
Wnt signaling pathway activation. The canonical Wnt signaling pathway: In the Wnt-off state, Gsk3β, and Apc phosphorylate β-catenin and degrade it by ubiquitination. In the Wnt-on state, the canonical Wnt binds to Fzd receptors and Lrp5/6 co-receptors. This interaction recruits Dvl and Axin to the Wnt–receptor complex, and causes stabilization of β-catenin in the cytosol. Next, β-catenin is translocated into the nucleus, where it binds to the Tcf/Lef regions and activates Wnt target genes. Non-canonical Wnt signaling pathways: In the calcium pathway, binding of non-canonical Wnt ligands to Ror-Ryk-Fzd recruits Dvl which, in turn, binds to small GTPase to further activate phospholipase C (PLC). In the PCP pathway, non-canonical Wnt ligands bind to the Ror/Ryk-Fzd receptor complex, recruiting Dvl to the plasma membrane and activating Rac and Daam1. Next, target genes are transcriptionally activated through JNK and MAPK. Created with BioRender.com.

Wnt signaling is an evolutionarily conserved signaling pathway that controls a wide range of biological responses, including proliferation, differentiation, preservation of the stem cell pool, control of lineage-specific tissue differentiation during embryogenesis, and maintenance of adult tissue homeostasis [3,4,5]. The Wnt pathway is divided into two main groups—i.e., β-catenin-dependent (canonical) and β-catenin-independent (non-canonical)—which can be further divided into the planar cell polarity (PCP) and the Wnt/Ca^2+^ pathways (Figure 1). The canonical Wnt cascade is inactive in the absence of Wnt ligands, and this leads to phosphorylation of β-catenin by a cytoplasmic multiprotein complex that contains the kinases glycogen synthase kinase 3β (Gsk3β) and casein kinase 1a (Ck1a), the scaffold protein Axin, and adenomatous polyposis coli (Apc) [26,27]. This phosphorylation targets cytoplasmic β-catenin for degradation by the ubiquitin–proteasome system. Canonical Wnt signaling is activated by binding of Wnt ligands to the membrane receptor Frizzled (Fzd) and the co-receptor low-density lipoprotein-receptor-related protein (Lrp) 5/6. Formation of the Wnt–receptor complex leads to the recruitment of the core components of the destruction complex to the cell surface, phosphorylation of the cytoplasmic tail of Lrp6 by Gsk3β and Ck1α, and stabilization of β-catenin in the cytoplasm and its nuclear translocation. In the nucleus, β-catenin interacts with the T-cell factor/lymphoid enhancer factor (Tcf/Lef) family of transcription factors, and regulates the expression of target genes [28,29]. The PCP pathway was originally described in the fruit fly *Drosophila melanogaster*, and controls coordinated, uniformly polarized cellular behavior in a wide variety of cells [30]. In mammals, PCP regulates key developmental processes ranging from neural tube closure to determination of left–right (L–R) asymmetry, and demonstrates essential roles in vertebrate development [31]. In the PCP pathway, the non-canonical Wnt ligands interact with the receptor Fzd and co-receptors (receptor tyrosine kinase-like orphan receptor (Ror)/receptor tyrosine kinase-related tyrosine kinase (Ryk)/protein tyrosine kinase 7 (Ptk7)). These interactions regulate the small GTPase molecules Rho, Rac, and Cdc42, and activate the kinases c-Jun N-terminal kinase (Jnk), the mitogen-activated protein kinase (MAPK) pathways, and Rho/Rho-associated coiled-coil-containing protein kinase (Rock) to control cell polarization and migration [32,33,34]. In the Wnt/Ca^2+^ pathway, intracellular Ca^2+^ is activated by the binding of Wnt to Fzd and coupling between Fzds and G proteins. This further activates protein kinase C (PKC), calcium/calmodulin-dependent protein kinase type II (CaMKII), and nuclear factor of activated T cells (NFAT), and regulates cell movement, cell fate, and cell migration as well as suppressing the canonical Wnt pathway (Figure 1) [33,34,35]. 

**Figure 2:**
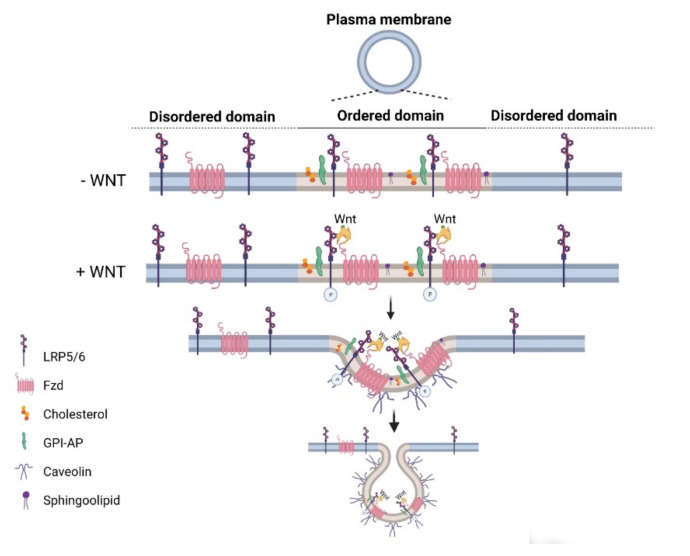
Canonical Wnt signaling activity is controlled by components of the plasma membrane. In general, the ordered domains are enriched in cholesterol, glycolipids, and caveolin. The ordered domains are necessary for the binding of the canonical Wnt ligand to its (co)receptors, Lrp5/6 phosphorylation, receptor endocytosis, and signaling activity. Created with BioRender.com.

Wnt pathways are fine-tuned by a number of positive and negative regulators that can affect the ligand–receptor complex interactions at the plasma membrane, cytoplasmic events, or nuclear control of transcription [22,36,37,38,39,40,41,42,43]. The plasma membrane plays key roles in protection of the cell from its surroundings, providing a stable environment inside the cell, management of molecular transport, and cell–cell communication. Embodying numerous receptors and lipids that take part in cell signaling, the plasma membrane is critical for the reception of signals and their transmission through a series of molecular switches to internal signaling pathways. The activity of the canonical Wnt signaling pathway is also dependent on the membrane components that tightly regulate the interaction of ligands with their (co)receptors in the specialized membrane nanodomains, i.e., the ordered membrane domains or lipid rafts (Figure 2). The ordered domains are necessary not only for the proper interaction of the canonical Wnt ligand with its (co)receptors, phosphorylation of Lrp6, endocytosis of receptor complexes, and downstream canonical signaling activity, but also for the regulation of non-canonical Wnt signaling activity [15,42,44,45]. The roles of the ordered membrane domains in the activation of Wnt pathways have been reviewed in detail previously [22]. Here, we focus on the involvement of Wnt–receptor complex components and ordered membrane domains or lipid rafts in certain brain disorders and metabolic diseases. 

## 3. Wnt Signaling at the Plasma Membrane in Aging and Brain Disorders

Synaptic plasticity and transmission are reduced in the aging brain [46,47]. Loss of synaptic contact is a major feature of Alzheimer’s disease (AD), which is the most common cause of dementia [48]. Late-onset Alzheimer’s is the most common form of the disease, and the decrease in synaptic strength is closely associated with the reduced susceptibility of synapses to toxic molecules such as Aß [46]. Wnt signaling is known to play essential roles in synapse formation, function, and maintenance in the adult brain [49]. The Wnt signaling pathway also appears to be associated with replicative cellular senescence and aging [46]. The fact that the Wnt signaling pathway is disturbed during aging suggests that Wnt signaling can enhance synaptic function during aging and improve AD-related synaptic pathology. Expression of the plasma membrane components Wnt2b, Wnt6, Wnt7a, Fz2, and Fz3 has been found to decrease with age, while that of Lrp6 and the Wnt antagonist secreted frizzled-associated protein 1 (Sfrp1) increase during aging. [50]. Moreover, there is a general decrease in Wnt signaling with age—especially in the lungs and the brain [51]. Interestingly, long-term loss of 17β-estradiol (E2 or estrogen) after menopause causes an elevation of neural Dickkopf-1 (Dkk1)—a secreted Wnt antagonist that binds to Lrp6—and a reduction in Wnt/β-catenin signaling activity, ultimately causing neurodegeneration [52]. Moreover, E2 has been shown to suppress Dkk1 and employ a neuroprotective effect. Thus, estrogen appears to prevent neurodegeneration through Wnt/β-catenin signaling activation.

The dynamic alterations in plasma membrane domains are considered to be important for signal transduction in neurogenesis and, thus, contribute to aging and the development of brain diseases. A prominent work has supported this hypothesis by showing that enrichment of the (pro)renin receptor ATP6AP2 in caveolae/lipid raft microdomains is essential for neuronal differentiation of stem cells, with a concomitant transition from Wnt/β-catenin to Wnt/PCP signaling, and that these domains may be used as a potential target for the treatment of neurodegenerative disorders [53]. In a group of patients with neurodegenerative diseases, ATP6AP2—which is associated with membrane microdomains—has been reported to be regulated by intracellular Ca^2+^ and Gαq proteins, and to induce neuronal differentiation [53]. The glycoprotein M6a (GPM6a), which becomes localized to the lipid rafts and induces their clustering in a palmitoylation-dependent manner, likewise regulates neuronal polarity and accelerates neuronal differentiation [54]. Palmitoylated membrane proteins generally prefer to concentrate in lipid rafts [55,56,57]. Since GPM6a is one of the major palmitoylated proteins in the adult brain, it is likely that the lipid rafts play key roles in brain functions, and are associated with brain diseases including AD, PD, SZ, and Huntington’s disease [54,58]. Under this section, we will review dysregulation of the Wnt signaling pathway at the plasma membrane and its domains in the common brain disorders AD, PD, and SZ.

### 3.1. Alzheimer’s Disease

Alzheimer’s disease (AD) is a neurodegenerative disorder that accounts for two-thirds of dementia cases [59,60,61]; it is defined as an irreversible and progressive brain dysfunction, and characterized by deterioration or loss of cognitive functions such as memory and thinking and, at the most advanced stage, of the ability to carry out daily tasks. The pathology of the disease is characterized by accumulation of certain proteins, inflammatory changes, and neuronal cell death [48]. The extra- and intracellular accumulation of amyloid-beta (Aβ) plaques and hyperphosphorylated Tau protein correlate strongly with cognitive impairment [60,62]. In addition, loss of synapses is observed in the early stages of AD progression, while neuronal cell death is observed in the late stage [63,64].

The secreted Wnt antagonist Dkk1 was found to be highly expressed in the brains of Alzheimer’s patients and in murine AD models [65,66]. In parallel, increased Gsk3ß activity, reduced cytoplasmic ß-catenin levels, and low Wnt signaling activity were detected in the brains of AD patients [67,68,69,70,71]. Mass spectrometric analysis of samples obtained from patients’ brains also validates the decrease and deterioration in canonical Wnt signaling [72,73,74]. Aß accumulation in the hippocampal neurons is known to reduce canonical Wnt activity and enhance synaptic loss by increasing Dkk1 expression [75]. Inhibition of Dkk1 by neutralizing antibodies has been found to completely abolish the Aß effect on synapses and prevent synaptic loss [76]. In a transgenic murine model that expresses Dkk1 in the brain in an inducible manner, Dkk1 was found to cause synapse and memory deficits in the striatum and hippocampus, decrease in long-term potentiation (LTP), and increase in long-term depression (LTD), without affecting cell viability [77,78]. Moreover, postnatal deletion of Lrp6 from forebrain neurons in a murine model of AD triggered amyloidogenesis of APP, leading to synaptic loss and exacerbating AD pathology [79]. Dkk1 also acts as an activator of non-canonical Wnt–PCP signaling and, hence, promotes synapse withdrawal and further Aβ production [73]. 

Genetic variants of Lrp6 have been investigated for being a risk factor for late-onset AD. For example, the Ile-1062-Val variant (exon 14) of Lrp6, which reduces the activity of Wnt/β-catenin signaling, appears to be a genetic risk factor for AD [68]. Moreover, silencing of long non-coding RNA (lncRNA) SOX21-AS1, which targets FZD3/5 genes, causes activation of the Wnt/β-catenin pathway, reduces neuronal oxidative stress, and suppresses neuronal apoptosis in mice with AD [80]. Activation of canonical Wnt signaling is known to protect the hippocampal neurons against the neurotoxicity of Aβ peptides [46]. The canonical pathway ligand Wnt3a and receptor Fzd-1 inhibit Aβ toxicity by activating the Wnt/β-catenin pathway [81,82]. In conclusion, activation of the Wnt/β-catenin pathway reduces Aβ formation and neural toxicity, leading to synapse activation. 

In addition to the protein components of the Wnt pathways, the lipid membrane microenvironment also plays key roles in AD. For example, cholesterol content and distribution are associated with Aβ production and cell dysfunction in AD [20]. Aβ appears to accumulate in the lipid rafts, which act as the primary mediators of the relevant oxidative stress at the plasma membrane [83]. Amyloid peptides tend to bind to the membranes specifically within the ordered domains enriched in cholesterol [84,85,86,87]. Moreover, Aβ was found to bind to the sphingolipid GM1 gangliosides (GM1/Aβ) in the brains of patients who exhibit early pathological features of AD, suggesting that GM1/Aβ may promote amyloid toxicity [88]. This binding of Aβ to the plasma membrane has also been found to be facilitated by the cholesterol content of the membrane, by altering the binding capacity [89]. Clustering of GM1 appears to be strongly enhanced by another sphingolipid, sphingomyelin—particularly at the neuritic terminals [90]. These findings correlate with the significantly increased SM levels in the membrane microdomains and synaptosomes that are isolated from aged murine brains [91]. In contrast, a more recent work has demonstrated that while sphingomyelin triggered oligomerization of Aβ monomers, physiological levels of GM1 did not [92]. Thus, decreasing levels of GM1 in the brain can reduce protection against Aβ oligomerization and contribute to the onset of AD. In this case, the oligomerization-promoting action of GM1 can be explained by the extreme and non-physiological experimental conditions [92]. Thus, the influence of GM1 along with other sphingolipids in Aβ accumulation deserves to be further explored. Since regulation of the Wnt pathway has been widely associated with the ordered domains of the plasma membrane, it is absolutely essential to investigate the influence of changes in the membrane lipid environment on amyloidogenesis via affecting Wnt signaling activity. This would unravel yet-unknown mechanisms of Wnt signaling in AD progression, and propose potential new therapeutic approaches.

Although Aβ accumulation and abnormal Tau protein accumulation are the most widely accepted mechanisms for AD, they are insufficient to explain the disease mechanism and to target AD therapeutically [60,61]. For example, in clinical studies conducted to date, reducing Aβ alone has not given promising results. Cholinergic neurotransmission in the cerebral cortex and basal forebrain has been shown to play an important role in the development of AD, proposing the cholinergic system as a main focus in the treatment of the disease [93]. Nicotinic acetylcholine receptors (nAChRs)—the ligand-gated ion channels that respond to the neurotransmitter acetylcholine—reside and cluster in the lipid rafts and interact with lipids surrounding the transmembrane domain [20,94]. The changes in levels of cholesterol and sphingomyelin at the plasma membrane can alter the localization and function of the nAChRs. Disruption of lipid rafts in rat primary hippocampal neurons by targeting the levels of cholesterol and sphingomyelin results in significant changes in nAChRs [95]. Moreover, agrin—a proteoglycan that acts at the neuromuscular junction—mediates AChR clustering in the lipid rafts, causing further partitioning of the muscle-specific receptor tyrosine kinase (MuSK) into lipid rafts [94]. Lipid rafts are necessary for MuSK activation and downstream signaling. Interestingly, AChR clustering is mediated by rapsyn—an intracellular protein that constitutively becomes localized to the lipid rafts, and is dependent on the rafts to interact with the AChR [94]. These studies reveal that lipid rafts considerably affect the function of the nAChRs, which have been shown to play a crucial role in AD development, and further point to the importance of lipid rafts in the development of therapeutic approaches for AD.

### 3.2. Parkinson’s Disease

Parkinson’s disease (PD) is the second most common neurodegenerative disease, following AD, with an age-dependent prevalence of 1-4% [96,97,98]. PD is a sporadic or familial inherited disease with complex symptoms that include resting tremor, bradykinesia, increasing muscle tension, and postural instability [96,97]. PD is characterized at the molecular level by loss of dopaminergic neurons in the substantia nigra—a region in the midbrain—and accumulation of ubiquitin- and α-synuclein (aSYN)-positive cytoplasmic inclusions called Lewy bodies (LBs) [99,100]. Mitochondrial dysfunction, protein misfolding and aggregation, oxidative stress, immunity inflammation, autophagy, and apoptosis have been shown to contribute to neurodegeneration in PD [101]. Glutamate excitotoxicity also plays a role in the pathogenesis of PD, and excitatory amino acid transporters (EAATs) are important in removing glutamate [102]. The Wnt signaling pathway has been reported to exert a protective effect against PD by inducing the expression of EAATs [103]. 6-Hydroxydopamine treatment promoted cell death in astrocytes and dopaminergic cells, and inhibited expression of Wnt1, β-catenin, and EAAT2; on the other hand, Wnt1 overexpression decreased glutamate levels, and upregulated β-catenin, EAAT2, and nuclear factor kappa-Β (NF-κB) levels [103]. Thus, by promoting EAAT2 expression, Wnt1 could inhibit dopaminergic neuron loss and play a cytoprotective role in PD.

The pesticides paraquat and maneb, which interfere with mitochondrial function and cause toxicity via oxidative stress, have been shown to cause neurotoxicity in the dopaminergic system and, thus, increase the risk of PD [104,105]. Both toxins decrease expression of Wnt1 at the protein and mRNA levels in rats, while increasing expression of Wnt5a, which induces differentiation of neural cells into dopaminergic precursors, and increases proliferation of progenitor cells [106]. Moreover, miR-34-b/c, which was found to be downregulated in brain areas of PD patients prior to the appearance of motor dysfunction, silences expression of Wnt1 by targeting it at the 3’UTR, and enhances differentiation of murine embryonic stem cells or transdifferentiation of fibroblasts into dopaminergic neurons [107,108]. In contrast, Wnt4 overexpression in a *Drosophila* model of PD has been found to significantly reduce disease-related abnormalities, such as impaired flight ability, by inhibiting autophagy and apoptosis and restoring mitochondrial function [109]. These data, taken together, suggest that different Wnt ligands could play opposing roles—i.e., offensive or protective—in the course of PD [109].

Properties of the membrane lipid environment can affect the prognosis of PD. The familial PD-linked proteins α-synuclein, LRRK2, parkin, and DJ-1 have been demonstrated to be associated with the lipid rafts, strongly suggesting that lipid rafts are involved in the pathogenesis of PD [110,111]. E3 ubiquitin ligase tumor necrosis factor-receptor associated factor 6 (TRAF6)—which binds to and ubiquitinates mutant DJ-1 and aSYN proteins, stimulates the aggregation of these insoluble and polyubiquitinated forms as LBs in PD [112]. Colocalization of TRAF6 with aSYN in LBs in postmortem brains of PD patients highlights the importance of atypical ubiquitination in the pathogenesis of PD. Strikingly, the majority of the endogenous TRAF proteins were detected in the lipid raft fractions, and this was controlled by the RANK ligand—the receptor activator ofthe NF-κB ligand [113]. Furthermore, GM1 levels have been found to increase in some neuronal populations in PD, and elevated GM1 levels are associated with increased toxicity of misfolded protein oligomers [114]. aSYN directly associates with the ganglioside GM1, which is highly abundant in the lipid rafts, and results in the elimination of aSYN fibrillation by supporting its internalization [115]. Thus, there is growing evidence that both Wnt signaling and plasma membrane domains are associated with the pathogenesis of PD. However, the potential link between Wnt signaling and PD through membrane domains remains to be investigated. It would be very interesting to test the potential of membrane microdomains as therapeutic targets in PD.

### 3.3. Schizophrenia

Schizophrenia (SZ) is a chronic brain disorder and severe mental disorder that is characterized by aberrant neural lamination and orientation in the hippocampus. Patients with SZ interpret reality abnormally, and are characterized by extremely disorganized and abnormal motor activity, behavioral variability, delusions, disorganized thinking, and hallucination [116,117]. Neurodevelopmental abnormalities such as abnormal brain development, improper neuronal migration, altered spatial neuronal arrangement, and absence of gliosis have been reported in SZ [118]. Given the key roles of Wnt signaling in the development of the nervous system, it is not surprising that misregulation of Wnt signaling has numerous deleterious effects on neural development, and thereby contributes to the pathogenesis of neurodevelopmental disorders. Owing to its connection with dysregulations of nervous system development—particularly synapse formation and maintenance—the pathogenesis of SZ has been associated with abnormal Wnt signaling [119,120,121]. A change in the level of the neurotransmitter dopamine (DA), which has been associated with many physiological processes in the central nervous system (CNS), has been implicated in SZ [122]. Canonical Wnt/β-catenin signaling plays a key role in controlling DA activity in neuronal fate decision. More recently, GSK3-β inhibition and β-catenin stabilization were found to promote the transformation of neural precursors into dopaminergic neurons [123]. Levels of β-catenin, APC, and GSK-3 were found to be altered in the hippocampi of schizophrenic (DSMIIR criteria) samples or murine models when compared to controls [124,125,126]. Expression levels of the ligand Wnt1 and the receptor FZD7 were increased, while those of the Wnt antagonists Dkk-1, Dkk-3, Dkk-4, and sclerostin were reduced in SZ patients, resulting in inhibition of the canonical pathway and activation of the non-canonical pathway [127,128,129,130]. Moreover, genes in the non-canonical ligand Wnt5a signaling network have been identified as becoming altered in SZ [131]. Genome-wide single-nucleotide polymorphism analysis has revealed FZD1 as a gene associated with SZ [132]. While FZD3 has also been proposed as a potential SZ susceptibility locus [133,134,135], genetic linkage studies have failed to support any major contribution of FZD3 to SZ susceptibility in the general population [136,137]. These findings suggest that Wnt signaling can be a targetable pathway for the treatment of SZ.

During the development of SZ, lipid homeostasis in the CNS also affects the fatty acid content in lipid rafts. Dietary supplementation of unsaturated omega-3 fatty acids causes their incorporation into the neural cell plasma membrane, increases membrane permeability, and modifies the organization of the lipid rafts [138,139]. These changes modify the GPCR activity in lipid rafts and the neural conduction [138]. The ordered-domain-preferring sphingomyelins were found to be decreased in SZ patients [140]. Consequently, the composition of the lipid membrane structure can affect the clustering and function of signaling pathways—including Wnt signaling—in SZ patients. As a result, Wnt signaling pathways and the membrane lipid environment can together be considered to be promising points that deserve consideration, so as to better understand the pathology of aging and brain disorders at the mechanistic level, and propose novel therapeutic approaches.

## 4. Wnt Signaling Pathway in Metabolic Diseases 

Wnt signaling is a major regulator of the development and growth of various tissues and organs involved in bodily metabolism, relating it with a range of metabolic diseases including diabetes, obesity, NAFLD, and NASH, which will be discussed here.

Nonalcoholic fatty liver disease (NAFLD) is the most common cause of chronic liver disease, which begins with isolated steatosis and advances to nonalcoholic steatohepatitis (NASH), steatofibrosis, and cirrhosis. 

### 4.1. Diabetes and Obesity

Several in vitro and in vivo studies have shown that the components of the Wnt signaling pathway are involved in β-cell proliferation, insulin secretion, and lipid metabolism [141,142,143]. Moreover, Wnt/β-catenin is linked to the long-term complications of type 2 diabetes mellitus (T2DM) and nephropathy [144,145]. T2DM is the most common type of metabolic disease [146]. The findings of recent genome-wide association studies in humans have identified *TCF7L2*/*TCF4* as a susceptibility gene for T2DM, and associated various *TCF7L* polymorphisms with a significantly higher risk of developing T2DM [147,148,149]. Secreted Wnt6 contributes to diabetes-associated centrosome amplification by activating the canonical pathway via the Fzd4 receptor [150]. Wnt signaling inhibition using Dkk1 significantly decreased neovascularization in diabetic rats as compared to untreated diabetic rats [151]. Furthermore, mutations in the Wnt signaling pathway’s components have been reported in patients with proliferative diabetic retinopathy—an advanced eye disease seen in diabetic people [151]. The Wnt signaling proteins Fzd4, TSPAN12, NDP, Lrp5, Lrp6, and β-catenin were also elevated in diabetic retinopathy in humans and animal models [151,152]. 

Due to the potential functions of the Wnt/β-catenin signaling pathway on bone development and remodeling, its dysregulation in T2DM makes patients more susceptible to bone complications. Sclerostin, which antagonizes the Wnt/β-catenin pathway by binding to Lrp5/6, is a small protein expressed by the *SOST gene* in osteocytes, and was found to be expressed at higher levels in T2DM subjects than in controls [153,154]. Interestingly, T2DM patients had lower levels of bone turnover markers and β-catenin, which are negatively correlated with sclerostin, suggesting that sclerostin prevents bone turnover by suppressing the canonical Wnt signaling pathway [153]. 

Vascular calcification is one of the most common complications in patients with T2DM. miR-128-3p accelerates cardiovascular calcification and insulin resistance in T2DM rats by targeting the pancreatic islet endocrine cell marker ISL-1 and activation of the Wnt pathway [155]. Upregulation of miR-128-3p enhanced the expression of Wnt1, β-catenin, and GSK-3β at the transcriptional level, and also increased phosphorylation of β-catenin and GSK-3β [155]. The Wnt/β-catenin pathway has been shown to be activated by another microRNA—miR-27a—which suppresses the Wnt antagonist Sfrp1 and activates Wnt/β-catenin signaling to promote the occurrence of renal fibrosis in diabetic nephropathy [156]. These findings collectively reveal the Wnt signaling pathway as a promising target for the treatment of T2DM and associated diseases.

The Wnt/β-catenin signaling pathway plays a vital role in adipose tissue lipogenesis and adipocyte metabolism—particularly under obesogenic conditions [157,158]. A cohort study involving 1004 people with atherosclerosis found that expression of Wnt5a was elevated in adipose tissue, with a concomitant increase in its receptors Fzd2 and Fzd5 in the human arterial wall and in vascular oxidative stress due to activation of NADPH oxidases [159]. Mice homozygous for the Lgr4 mutation, which acts as the receptor for the Wnt agonist R-spondins (Rspos) to enhance canonical Wnt signaling, showed reduced adiposity and resisted obesity [160]. These mice exhibited a parallel increase in energy expenditure of brown-like adipocytes in white adipose tissue, counteracting obesity. Moreover, a functional low-frequency missense variant of Lgr4 has been associated with an increased risk of obesity [160]. Adiponectin is an adipose-tissue-derived adipokine, and its levels are reduced during obesity [161,162]. AdipoRon, a small-molecule adiponectin receptor agonist, suppresses Rspo1-mediated canonical Wnt signaling [163]. By decreasing the free cholesterol levels at the plasma membrane, redirecting the cholesterol into the lysosomes, and reducing membrane rigidity, AdipoRon modulates the membrane order and inhibits Wnt signaling [163]. Therefore, it is likely that obese individuals, with low levels of circulating adiponectin, will exhibit increased plasma membrane rigidity and elevated Wnt signaling activity.

### 4.2. Nonalcoholic Fatty Liver Disease (NAFLD) and Nonalcoholic Steatohepatitis (NASH)

The liver has critical functions in the control of metabolic pathways such as glucose metabolism and fatty acid metabolism [164]. The Wnt signaling pathways regulate fatty acid metabolism, and epigenetic activation of the canonical Wnt signaling pathway has been associated with a fat metabolism disorder called NAFLD [165]. NAFLD, defined as a range of conditions caused by the accumulation of fat in the liver, is one of the most common causes of chronic liver disease, which begins with steatosis, advances into NASH, and further progresses to end-stage liver diseases such as fibrosis, cirrhosis, and hepatocellular carcinoma (HCC) [166]. The molecular mechanisms of NAFLD are poorly defined. Loss-of-function studies in the Wnt co-receptor Lrp6 have been associated with NAFLD. Mice homozygous for the Lrp6^R611C^ mutation exhibit both steatohepatitis and steatofibrosis features associated with NAFLD [167]. Impaired Wnt signaling in the homozygote Lrp6^R611C^ mice was efficiently remedied by administration of Wnt3a. Lrp6 knockdown also stimulated the non-canonical Wnt proteins RhoA (Ras homolog family member A) and ROCK2 (Rho-associated protein kinase), as well as their phosphorylated forms. Thus, the Lrp6 and non-canonical Wnt pathways are likely to be important therapeutic targets against NAFLD and NASH. The role of Lrp6 has also been evaluated in another study, where miR-21 was found to inhibit Lrp6 expression and Wnt β-catenin signaling activity, and enhanced the expression of critical lipid metabolic enzymes [168]. These results strongly suggest that targeting the Wnt β-catenin pathway at the plasma membrane can be an efficient therapeutic strategy against NAFLD and NASH.

Compositions of phospholipid fatty acids have been found to alter in the cell membranes of patients with T2DM, obesity, metabolic syndrome, or NAFLD [169,170,171,172]. The risk factors in NAFLD and NASH cause remodeling of the plasma membranes by changing their physicochemical properties. For example, co-exposure to the environmental contaminant benzo[a]pyrene and the hepatotoxicant ethanol triggered a general membrane order with higher lipid raft clustering in the plasma membrane of liver cells, and induced in vivo hepatotoxicity via membrane remodeling [173]. Moreover CD36—a scavenger receptor responsible for lipid accumulation and progression of metabolic dysfunction—localized more at the plasma membrane of hepatocytes in mice and humans with NASH [174]. Strikingly, inhibition of the palmitoylation of CD36 protected the mice from NASH by reducing the hydrophobicity of CD36 and reducing its localization at the membrane of hepatocytes [174,175]. Expression of Toll-like receptor 4 (TLR4), which is also involved in the pathogenesis of NASH, was found to be higher in the ordered membrane domains in NASH patients, and the TLR4 antagonist sparstolonin B attenuated TLR4 trafficking to these domains, as well as early liver inflammation in a murine model of NASH [176,177,178]. Co-expression of Fzd9 and Wnt3a with TLR4 in neuronal or glial cells as a response to inflammatory stimuli supports the idea that TLR4 trafficking in ordered membrane domains might also be controlled by Wnt signaling in fatty liver diseases [179,180]. Glucagon-like peptide 1 receptor (GLP-1R) likewise localizes in lipid raft/caveolae microdomains of the plasma membranes in liver samples of patients with NASH, and is transcriptionally activated by the canonical Wnt signaling pathway [181,182,183]. The Wnt/β-catenin pathway has also been shown to promote the activation of Nod-like receptor protein 3 (NLRP3) inflammasomes [184]. Strikingly, the activation of NLRP3 inflammasomes contributed to NAFLD and NASH, and they were further enhanced by palmitic acid in hepatic stellate cells [185].

Cholesterol appears to be another major actor that plays a role in the development of NASH. Dysregulation of hepatic cholesterol homeostasis causes accumulation of hepatic free cholesterol (FC) and oxidized low-density lipoprotein (oxLDL) in NAFLD and NASH [186,187,188]. The phytochemical curcumin suppressed expression of lectin-like oxLDL receptor-1 (LOX-1) via interruption of canonical Wnt signaling in hepatic stellate cells, which are the main effector cells of NASH-associated hepatic fibrogenesis [189]. Given the stimulatory role of Wnt signaling in cholesterol endocytosis/flux and the production of lipid droplets, and the importance of cholesterol in Wnt–receptor complex formation, further studies in NASH will unravel the potential role of Wnt signaling activation at the plasma membrane in the induction of hepatic FC levels [15,186,190].

## 5. Conclusions

The Wnt signaling pathways are essential for many cellular events that take place in development, homeostasis, and regeneration. Being the initiator of Wnt–receptor complex formation that activates the signaling pathway, the plasma membrane plays an essential role in the regulation of Wnt signaling. Signaling initiation and regulation strongly depend on the content and organization of the membrane. In pathological processes, including brain disorders and metabolic diseases, changes occur in the composition of membrane lipids and proteins. While the Wnt signaling pathway has been relatively better characterized in AD and PD with respect to the content and organization of the plasma membrane domains, there exists limited knowledge concerning this issue in SZ. Therefore, further studies are required in order to clarify the role of the Wnt signaling pathway in SZ in the context of plasma membrane organization. Understanding the molecular mechanism of the Wnt signaling pathway in the context of plasma membrane organization will contribute to the development of new therapeutic strategies for the diseases in which the Wnt signaling pathway is dysregulated.
